# Preparation and Characterization of Poly(lactic acid)-Based Poly(ethylene glycol) and Daphne Essential Oil-Loaded Smart Nanofibers for Thermal Protection

**DOI:** 10.1021/acsomega.3c07720

**Published:** 2024-10-11

**Authors:** Tugba GungorErtugral, Yalçın Coşkun, Ayhan Oral, Seyhan Ulusoy

**Affiliations:** † Department of Food Technology, Çanakkale Onsekiz Mart University, Çanakkale 17100, Turkey; ‡ Department of Plant Production and Animal Husbandry, Çanakkale Onsekiz Mart University, Çanakkale 17100, Turkey; § Department of Chemistry, Çanakkale Onsekiz Mart University, Çanakkale 17100, Turkey; ∥ Department of Biology, Suleyman Demirel University, Isparta 32260, Turkey

## Abstract

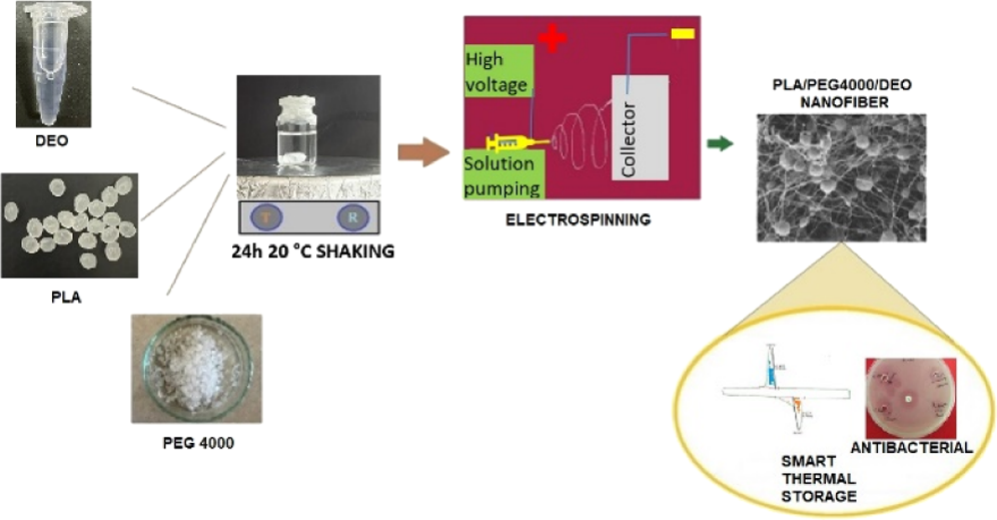

Phase change material (PCM) stores latent heat energy, and poly(ethylene glycol) (Mw: 4000) (PEG 4000) is also a solid–liquid PCM. PEG and poly(lactic acid) (PLA) polymers are biodegradable. Essential oils are known as plant extracts with antimicrobial properties. In this study, daphne essential oil (DEO) obtained by the distillation method and PLA/PEG/DEO composite nanofibers were prepared by the electrospinning method with PLA, PEG 4000, and daphne (*Laurus nobilis* L.) essential oil in certain ratios (100/100/20, 100/120/20, and 100/150/20). DEO showed an antibacterial effect against *Staphylococcus aureus* and *Escherichia coli* bacteria. Thermal behaviors of the nanofibers were characterized by differential scanning calorimetry and thermogravimetric analysis. Morphological features were observed by scanning electronic microscopy (SEM), crystal behavior by X-ray diffraction analysis, and molecular structures were examined by Fourier transform infrared spectroscopy. Essential oil composition was determined by GC–MS. The thermal decomposition temperatures of the nanofibers were found between 250 and 276 °C, and the latent heat storage energies of nanofibers were 69.06, 86.76, and 96.39 J g^–1^ at temperatures 59.0 and 54.37 °C. High PCM added fiber was observed as 182 nm diameter with 3.264 μm diameter spheres. The produced nanofiber matrix has the potential to be used in applications such as medicine, textile, and hot food logistics.

## 1 Introduction

The molecular weights of PEGs can vary between 200 and 35,000 g/mol, and they can be used in tissue engineering with their biodegradable properties.^1,2^ Phase change material (PCM) studies that focus on synthetic polymers and natural polymer studies are rare. PEGs are environmentally friendly during latent heat energy storage and utilization.^3^ They also provide thermal energy with their high latent heats of fusion and compatibility at melting/freezing limits; their noncorrosion or nondeterioration properties are functional for thermal storage systems.^4,5^ PCMs consist of paraffin, fatty acid, esters, and salt hydrates and their ternary mixtures. These properties allow for high latent heat energy storage during phase change and when ambient temperature decreases or rises, and they release heat energy they have stored to the environment.^6−8^ Also, 5 mm PCM plates can utilize latent heat energy for 4–8 h.^9^ Herbal treatments have very few adverse effects.^10^ Essential oil composition of fruits, leaves, and branches of daphne can exert antimicrobial effects against pathogenic and damaging bacteria and yeasts.^11^ Compounds contained in daphne essential oil (DEO) is capable of being drug candidates.^12^ Compounds within this oil, such as bioactive 1,8-cineol (eucalyptol) and methyl eugenol, can provide additional *Staphylococcus aureus* inhibitions along with bactericidal, antibacterial, and antistaphylococcal properties, which limit bacteria that cause oral infections and dental diseases.^13^ PLA has a linear structure that is produced from beet, sugarcane, and corn products and has a biocompatible structure obtained by lactic acid polymerization.^14^ Synthetic fibers were spun with electrical charge by Formhals in 1934,^15^ and PLA-based nanomaterials produced in this way could perform controlled release.^16^ PLA-based resin composites are also recommended for food packaging, biomembranes, and biomedical purposes.^17^ In addition to nanofibers prepared by synthesizing the PLA/PEG block copolymer and dissolving it in different solutions,^18,19^ there are also PLA/PEG nanofibers that are directly dissolved in solution environments and electrospinned by the electrospinning method.^20^ Pure PEG melting crystallization enthalpy is 175.50 J/g at 60.03 and 40.98 °C, but the PLA/PEG block copolymer has an enthalpy of 56 J/g between melting points of 44 and 55 °C, and the melting enthalpy value with the binding of PEG decreased.^21^ PLA/PEG nanofibers containing the active ingredient (Acyclovir) obtained by the blow spinning method have a melting enthalpy of 175 J/g at 257 °C.^22^ The latent heat energy storage capacity of PLA/PEG nanofibers may vary depending on the PEG density. With the goal of developing a new class of form-stable polymer–matrix phase change materials for thermal energy storage, cellulose acetate and poly(ethylene glycol) (PEG)-based composite fibers with five different molecular weight (Mn) grades were prepared via electrospinning. PEG/CA composite fibers were prepared by heating, and balanced thermal storage and release properties were determined in cooling processes. An increase in enthalpy occurred from 47.93 to 73.48 J/g with the increase in the molecular weight of PEG 6000 in PEG/CA composite fibers.^23^ PEG/PLA phase change composite fibers prepared by the electrospinning method using PEG 8000 had current heat storage capacity with melting enthalpy between 54.8 and 74.7 J/g. The decomposition temperatures of nanofibers were above 320 °C.^24^ In the polyethylene glycol (PEG)/epoxy resin composite, PEG serves as the latent heat storage material, and its latent heat reached 132.4 J/g, corresponding to the polyethylene glycol percentage of PEG/EP.^25^ Polyethylene glycol (PEG)/diatomite composite as a novel form-stable PCM for thermal energy storage differential scanning calorimetry (DSC) results showed that the melting temperature and latent heat of composite PCM are 27.70 °C and 87.09 J/g, respectively.^26^

There are some medical applications that require temperatures of 60 °C.^27^ Fascial tissue composed of collagen can contract when it reaches 60–80 °C.^28,29^ On the other hand, in the food industry, hot meals and ready meals are stored and served at 60 °C.^30^ It has also been shown that UV light and γ-radiation of PLA-based electrospun fiber membranes do not affect fiber morphology or alignment.^31^

PEG 4000 has a latent heat energy storage capacity of 167.6 J/g at 59.13 °C.^32^ There are studies on the production of PLA/PEG nanofibers, but this study differs from the literature by producing nanofibers that store latent heat energy at 60 °C and contain an antimicrobial agent (DEO). In this study, to prepare natural structure PCM nanofibers, they contain antibacterial essential oil and can store latent heat energy. The antibacterial properties of DEO obtained from daphne leaves against *Escherichia coli* and *S. aureus* were determined, and morphological structure, heat storage/release properties, and thermal degradation ranges of PLA/PEG 4000/DEO (100/100/20, 100/120/20, and 100/150/20) composite nanofiber with certain proportions were determined.

## 2 Materials and Methods

### 2.1 Materials

Poly(lactic acid) was supplied from 4043 D Nebraska, USA, Natureworks LLC (Mn = 160,000 g/mol), and analytical grade %99.8 *N*,*N*-dimethylformamide (DMF) and PEG (Mw: 4000 g mol^–1^) were purchased from Merck Company. Leaves of daphne were supplied harvested on September 17, 2022, in the Çanakkale Region of Türkiye, and DEO was the product obtained by the hydrodistillation method.

### 2.2 Methods

#### 2.2.1 DEO Distillation

Daphne leaves were cut into small pieces and added to a Clevenger where they were distilled for 3 h in 600 mL of distilled water. The ratio of essential oil’s wet weight was determined for 100 g. DEO samples were then dried in a desiccator with anhydrous Na_2_SO_4_ and stored in a capped tube wrapped in aluminum foil.^33^

#### 2.2.2 GC–MS Analysis of DEO

The composition of DEO following distillation was determined by a Shimadzu GC–MS QP-2010. Samples were kept at 100 °C for 5 min, and then the temperature was increased at a rate of 20 °C/min, followed by 10 °C/min, gradually increasing to 150, 200, and 240 °C. Readings were taken while increasing the temperature and when keeping it at 240 °C for 25 min (Table 1).^34^

**Table 1:** GC–MS Analysis Method of Daphnia Essential Oil

column	HP-88 100 m (length)–0.20 μm (thickness)–0.25 mm (diameter)
column oven temp	100 °C
injection temp	220 °C
injection mode	split
split ratio	30
carrier gas	he prim.pres. (500–900)
flow control mode	pressure
pressure	232.8 kPa
total flow	37.2 mL/min
column flow	1.10 mL/min
linear velocity	21.4 cm/s
purge flow	3.0 mL/min

**mass spectrometer**	
ion source temp	230 °C
interface temp	250 °C
solvent cut time	10 min

#### 2.2.3 Antimicrobial Testing of Daphnia Essential Oil

The antibacterial properties of DEO against *S. aureus* and *E. coli* bacteria were examined using the well diffusion method.^35^ For the well diffusion method, oil samples were poured into previously prepared Petri dishes as a second layer by adding 100 μL (0.5 Mc Farland) of overnight bacterial cultures to soft agar (Müller Hinton) containing 0.5% agar with dimethyl sulfoxide (DMSO). After the medium solidified, oil samples were diluted with DMSO and added to these wells with sterile Pasteur pipettes. After one night incubation, the inhibition zone diameters formed around the well were measured in mm, and their antibacterial effects were evaluated.^36^ DMSO was used as the negative control.

### 2.3 Preparation of PLA/PEG/DEO Nanofibers

PLA concentration was prepared with 48.8% w/v, and PLA, PEG 4000, and DEO ratios were 1:1:0.20, 1:1.2:0.20, and 1:1.5:0.20, respectively (Table 2). These solutions in 5 mL of DMF were stirred at 600 rpm for 26 h at 25 °C to dissolve. Prepared solutions were drawn into a 10 mL plastic syringe, placed in a pump compartment of an electrospinning device, and covered with aluminum foil. The electrospinning system (Inovenso LLC NE 100) ran at 17.8 kV, with a pacing of 0.62 mL/h, and the distance between the needles and the collector was 17.0 cm.^37,38^

**Table 2:** PEG 4000 Components of PLA/PEG/DEO Nanofibers

sample	PLA/PEG/DEO (mass ratio)	PEG content (%)	PLA content (%)
PCM1	100/100/20	44.44	44.44
PCM2	100/120/20	48.48	41.66
PCM3	100/150/20	53.33	37.04

### 2.4 Characterization of PCM Nanofibers

#### 2.4.1 Fourier Transform Infrared Spectroscopy Analysis

Fourier transform infrared (FTIR) spectroscopy analyses of composite nanofibers were performed with a 100 FTIR spectrum spectrometer in the transmission mode at 4 cm^–1^ resolution. The wavelength scanning range was between 4000 and 650 cm^–1^.

#### 2.4.2 Thermogravimetric Analysis

Thermal degradation investigations of prepared composite nanofibers were measured with thermogravimetric analysis (TGA) (SDT Q600 V20.9 Build 20), and the heating rate was 10 °C min^–1^ at between 0 and 650 °C in a nitrogen atmosphere.

#### 2.4.3 Differential Scanning Calorimetry Analysis

To determine thermal properties, composite nanofibers were analyzed using a DSC Q2000 V24.11 Build 124 device. The amount of sample prepared was around 10 mg and was measured between −20 and 100 °C, with a heating/cooling rate of 2 °C/min. An aluminum hermetic pan was used for measurements in a nitrogen atmosphere.

#### 2.4.4 Scanning Electron Microscopy Analysis

The morphological structures and some surface properties of PLA/PEG/DEO nanofiber composites were determined with a Carl Zeiss 300VP scanning electron microscopy (SEM) device. The accelerating voltage was 5 kV, and the samples were imaged by SEM after being coated with gold, thus increasing conductivity before imaging.

#### 2.4.5 X-ray Diffraction Analysis

X-ray diffraction (XRD) analysis of nanofibers was performed by using a PANalytical EMPYREAN (Cu K_α_, λ = 0.154 nm, 45 kV, 40 mA) was used for XRD analysis. The measurement was carried out at a 2θ angle of 3–80°, a scanning rate of 5°/min, and a scanning step of 0.02°.

## 3 Results and Discussion

### 3.1 GC–MS Analysis

The essential oil obtained by steam-distillation in the Clevenger system had a high fatty acid composition and contained a high rate of oleic acid, 68.41% (Table 3). Among the essential oil components defined in Table 4, eucalyptol, which is the major component, had the highest value with 41.98%, and additionally, 17.59% α-terpineol acetate, 10.36% β-myrcene, and 12.63% β-linalool were determined (Table 4).

**Table 3:** Fatty Acid Components of DEO

fatty acid components	(%)
palmitic acid (C16:0)	17.35
stearic acid (C18:0)	5.68
oleic acid (C18:1) (*n*–9)	68.41
linoleic acid (C18:2) (*n*–9, 12)	8.56

**Table 4:** Components of DEO

essential oil components	(%)
β-myrcene	10.36
d-limonene	2.62
γ-terpinene	2.64
α-terpinolene	0.28
p-cymene	1.37
β-linalool	12.63
β-elemene	0.31
α-farnesene	1.79
α-terpineol	3.01
α-phellandrene	0.59
eucalyptol	41.98
sabinene	1.42
(*E*)-sabinene hydrate	0.4
α-terpineol acetate	17.59
methyl eugenol	0.22
spathulenol	0.66

### 3.2 Antimicrobial Test of DEO

**Figure 1:**
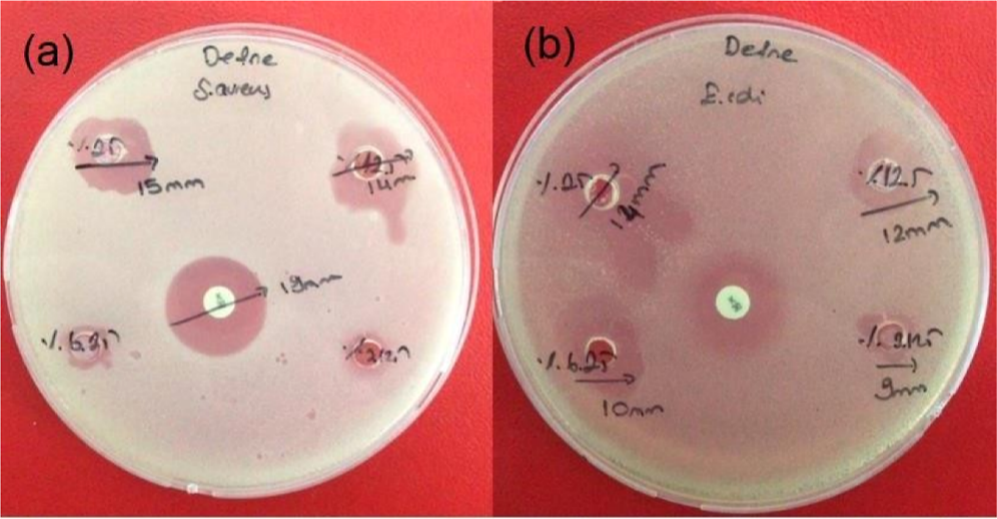
Antibacterial test of DEO for *S. aureus* (a) and *E. coli* (b).

The antibacterial activity of DEO was tested with the standard well diffusion method of CLSI. A zone with a diameter of 14–15 mm was observed in the DEO *S. aureus* test and a zone with a diameter of 12–14 mm in the *E. coli* test, and accordingly, it was determined to have an antimicrobial effect against *S. aureus* and *E. coli* bacteria (Figure 1a,b). A similar effect of eucalyptol has been reported against *E. coli* O157/H7 and *Staphylococcus typhimurium*.^39^

### 3.3 Morphology of PCM Nanofibers

**Figure 2:**
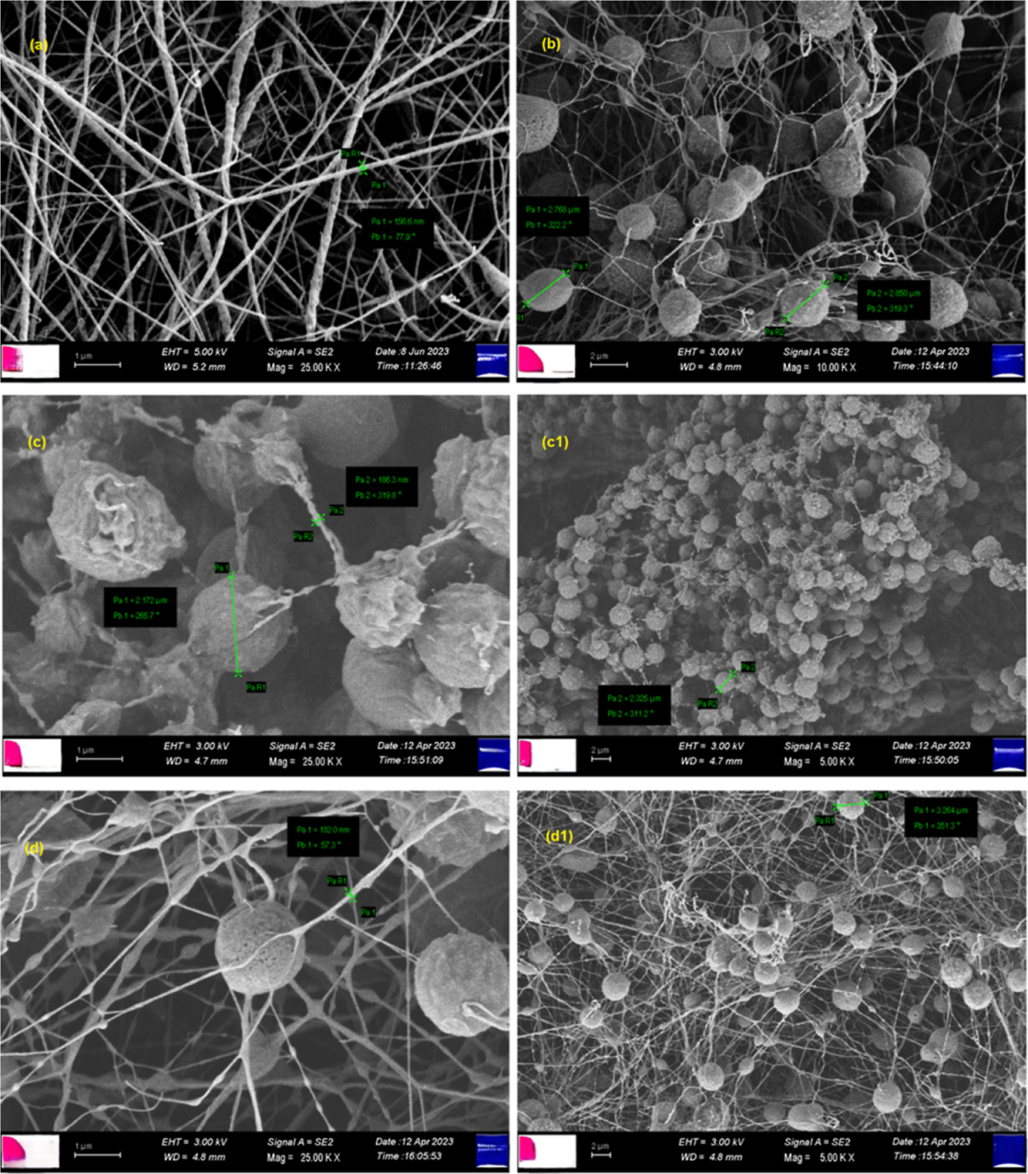
SEM images of (a) pure PLA; (b) PCM1; (c,c1) PCM2; and (d,d1) PCM3.

Images of prepared nanofibers and PLA (pure) were examined by SEM. It was observed from Figure 2a that the diameter of pristine PLA is on average 150 nm. The addition of PEG as PCM caused the diameters of the nanofibers to increase and resulted in the formation of beads in the nanofiber matrix. Diameters of the nanofibers are 186.3 and 182.0 nm for PCM2 and PCM3, respectively. PEG-inserted nanofibers also contained spherical structures with 2.8, 2.3, and 3.3 μm diameters in Figure 2b–d for PCM1, PCM2, and PCM3, respectively. Increasing the percentage of PEG caused the decreasing abundancies of the spherical structures.^37,40,41^

**Figure 3:**
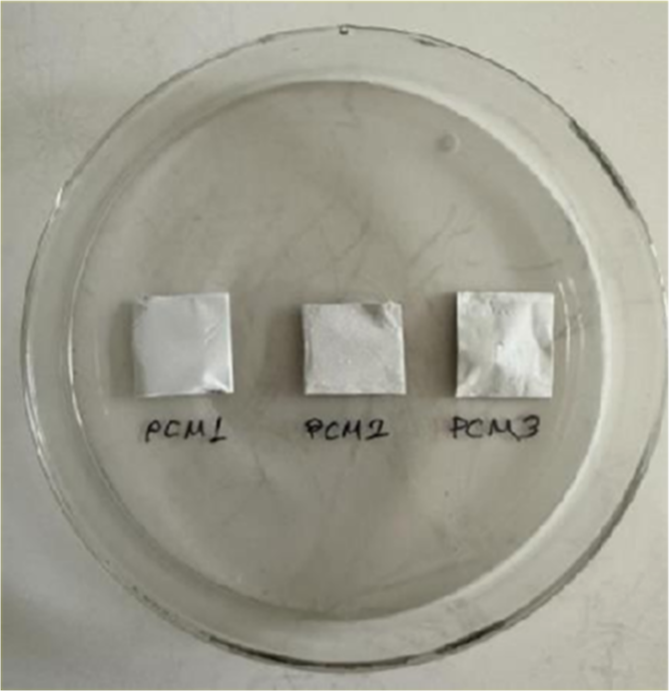
Photograph of PCM nanofibers on aluminum foil.

Nanofibers were electrospun on aluminum foil (Figure 3) and were separated from this foil and analyzed by SEM.

### 3.4 FTIR Analysis

**Figure 4:**
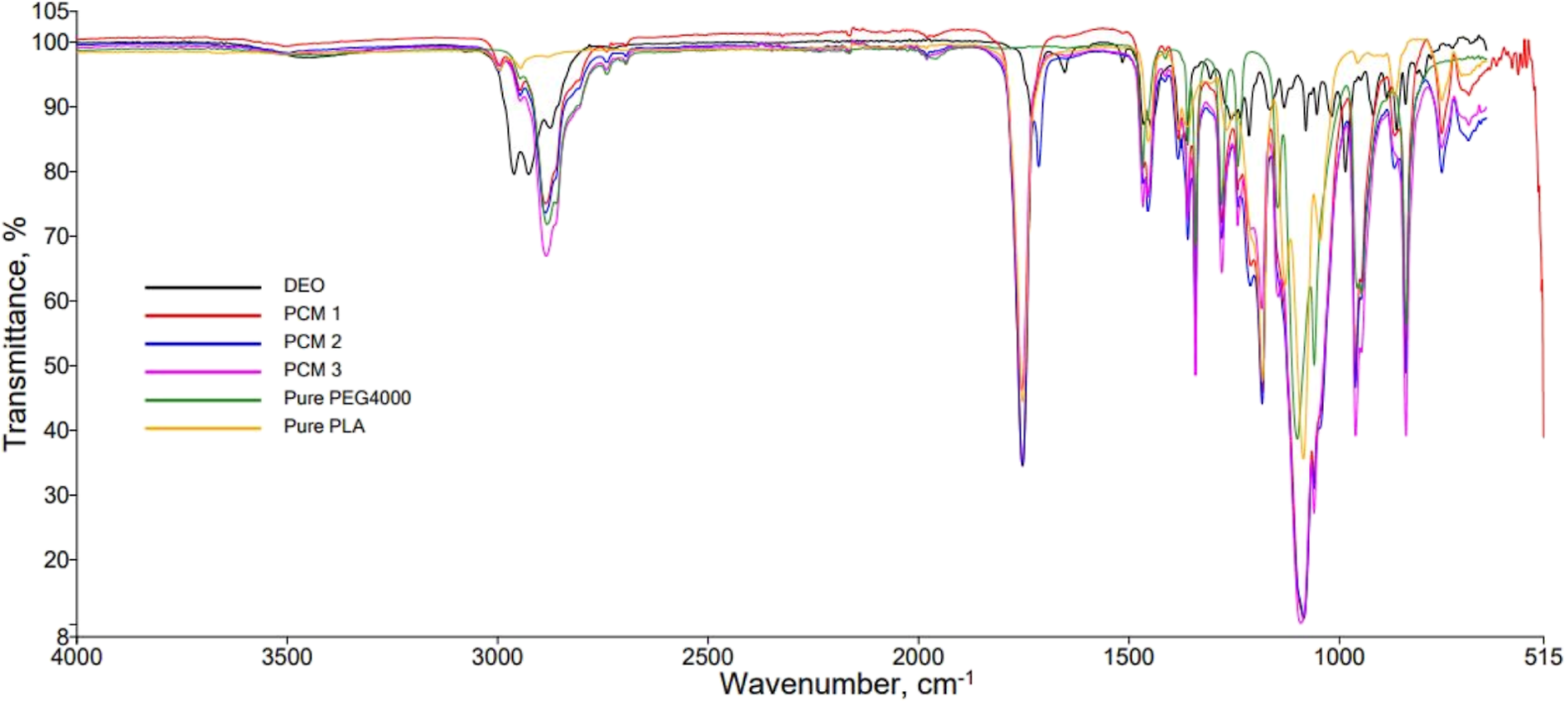
FTIR spectra of pure PLA, pure PEG 4000, DEO and PCM nanofibers.

FTIR absorption spectra of nanofibers were examined (Figure 4), and the C=O stretching vibration at 1752 cm^–1^ peak was observed for PLA. C–O stretching vibration was seen in peaks at 1084 and 1183 cm^–1^, and peaks characteristic of the C–H stretching vibration were around 2958 cm^–1^ and at 1436 and 1752 cm^–1^. The fact that the stretching vibration of C–H occurred at 1454 and 1752 cm^–1^ is an indicator of absorption. Stretching vibrational O–H of the PEG 4000 molecule was observed at 2886 cm^–1^. The spectrum belonging to the DEO showed a broad band with a maximum around 3500 cm^–1^ which was attributed to the OH stretching. O–H stretching vibration can be increased for the PEG 4000 ratio. From the DEO FTIR graph, the peaks at 1730 cm^–1^ and at 1640 cm^–1^ represent carbonyl stretching and C=C stretching, respectively. Bands at 1605, 1510, and 1460 cm^–1^ can be attributed to the aromatic ring in DEO contents. Bands observed 1375, 1210, and 1080 cm^–1^ can be associated with the C–O–C asymmetric and symmetric stretching.^24,42^ In addition, the increase in C–H and C–O–C stretches and shifts in the wave numbers of all characteristic bands of PEGs support the composite structure.

### 3.5 DSC Analysis

**Figure 5:**
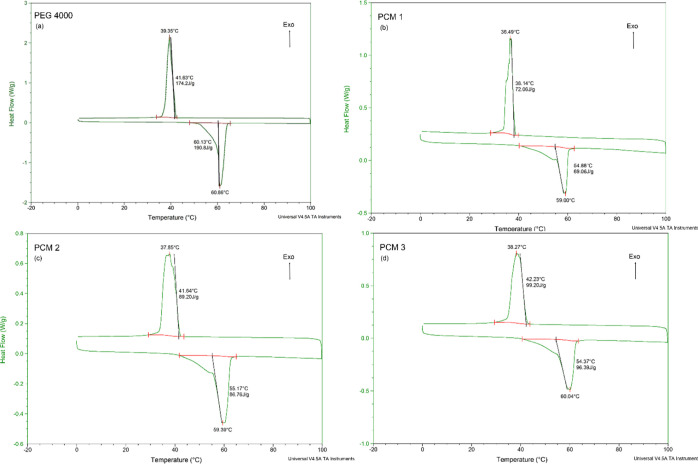
DSC curves of pure PEG 4000 (a), PCM1 (b), PCM2 (c), and PCM3 (d) nanofibers.

DSC curves of nanocomposites loaded with PEG 4000 were obtained, and the temperature range was between –20 and 100 °C at a heating rate of 2 °C/min (Figure 5). Enthalpy and temperature values of the nanofibers are shown in Table 5. According to DSC analysis results, pure PEG 4000 has a heat storage capacity of 190.8 J/g at a heat storage and release start temperature of 47.13 °C. Pure PLA stores 1.96 J/g latent heat energy at 56.71 °C,^38^ and nanofiber PCMs have higher enthalpies. The PCM1 composite nanofiber has a heat storage and release temperature starting point of 40.8 °C and a melting enthalpy of 69.06 J/g. While the initial phase change temperature for PEG 4000 was 47.13 °C and for PCMs this value decreased to an average of 40 °C, the melting enthalpy for PCM1 decreased from 190.8 to 69.06 J/g (Figure 5a). The melting enthalpy of the PCM2 nanofiber is 86.76 J/g at 59.39 °C. Among the nanofibers, the highest latent heat storage capacity is PCM3, with 96.39 J/g, and the highest PEG 4000 density (53.33%) (Figure 5d).^24,26^ The difference between melting and solidification enthalpy values was quite small, and there was no significant difference between melting (start of heating) and freezing points (start of cooling) of PCM samples.

**Table 5:** Phase Transition Temperatures and Latent Heats of Pure PEG 4000 and PCMs

melting			solidifying			
PCMs	melting point (°C)	*T*_peak_ (°C)	melting enthalpy (J/g)	freezing point (°C)	*T*_peak_ (°C)	freezing enthalpy (J/g)
pure PEG 4000	47.13	60.86	190.8	43.1	39.35	172.5
PCM1	40.8	59	69.06	40.5	36.49	72.06
PCM2	42.3	59.39	86.76	43.9	37.85	89.2
PCM3	40.7	60.04	96.39	42.23	38.27	99.2

### 3.6 Thermogravimetric Analysis

**Figure 6:**
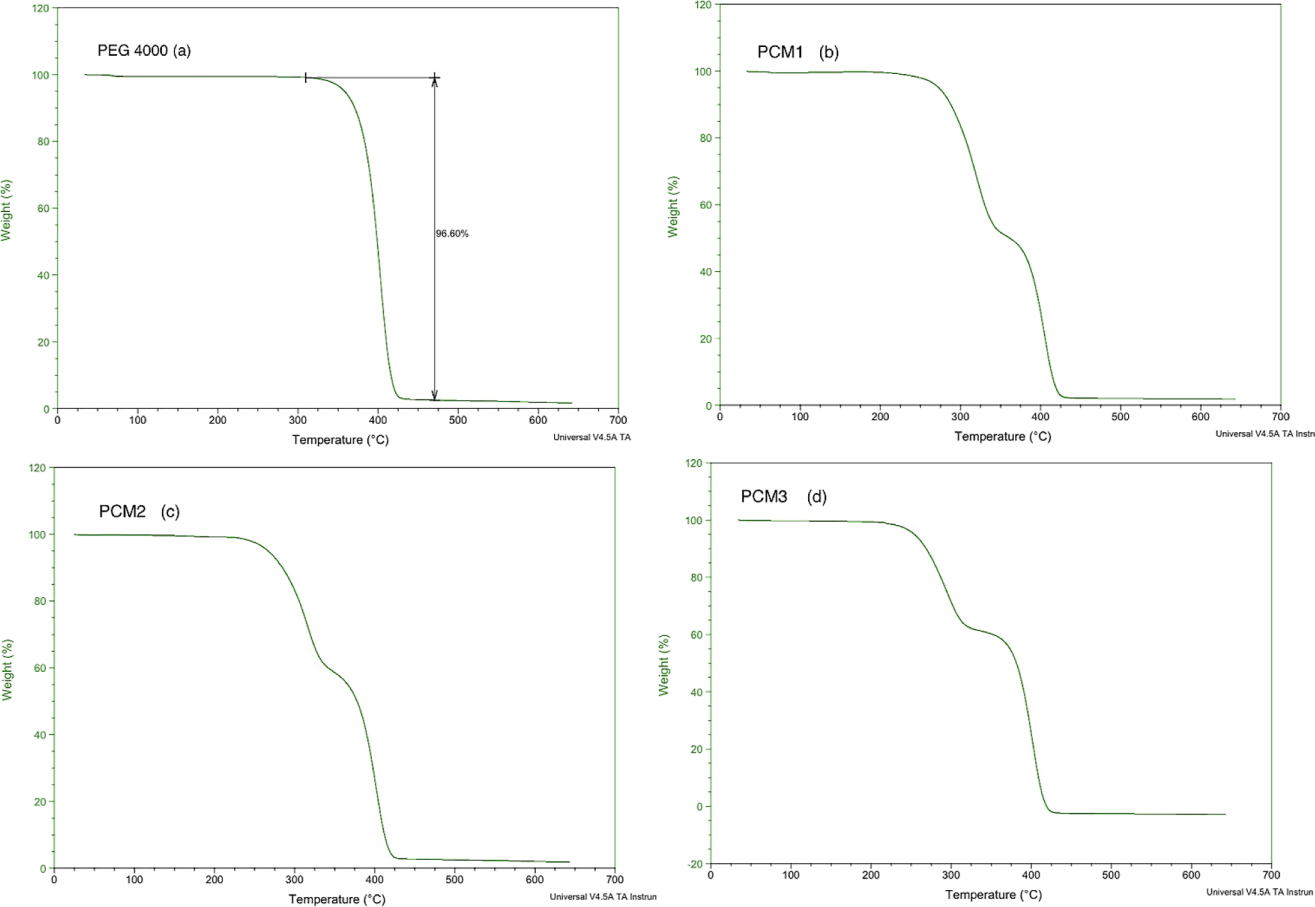
TGA curves of pure PEG 4000 (a) and PCM (b–d) nanofibers.

According to TGA results in Figure 6a, pure PEG 4000 showed degradation in one step and PCM1, PCM2, and PCM3 in two steps. Pure PLA degradation is around 300 °C, and thermal stabilities of PCM composites decreased with loaded essential oils in the polymer matrix.^43^ The TGA curve of PEG 4000 started to degrade at 218.7 °C, and PCMs were degraded in 2 steps according to changing maximum degradation temperature rate (Figure 6b–d). PCM1, PCM2, and PCM3 nanofibers showed degradation at temperatures of 200.8, 224.5, and 221.4 °C, respectively (Table 6). Produced PCM nanofibers can provide thermostability at 200 °C and lower applications, and morphological structure and thermal qualities of composite fibers may vary depending on PEG content.^24,44,45^ TGA results show that PEG-based PCMs degrade at high temperatures, and the presence of PLA and DEO did not significantly change the thermal stability of PEG 4000.

**Table 6:** Pure PEG 4000 and PCM Composite Nanofiber Thermal Degradation Results

samples	degradation interval (°C)		peak temperature (°C)
pure PEG 4000		218.7–424.2	374.8
PCM1	step 1	200.8–329.2	276.4
	step 2	330.5–424.7	376.7
PCM2	step 1	224.5–325.2	276.7
	step 2	332.7–423.4	376.2
PCM3	step 1	221.4–317.5	250.8
	step 2	324.2–423.6	374.3

### 3.7 XRD Analysis

**Figure 7:**
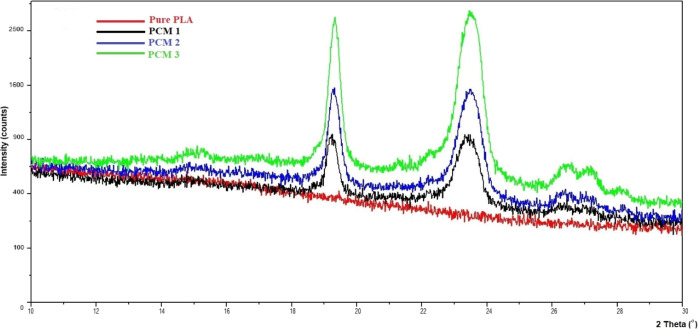
X-ray diffractograms of pure PLA, PCM1, PCM2, and PCM3 nanofibers.

X-ray diffractograms are shown in Figure 7. The peak which observed at 2θ = 15.41 is the characteristic peak of PLA; PLA nanofiber has not so high crystalline structure.^46,47^ PCM1, PCM2, and PCM3 have very strong and sharp crystalline peaks at 2θ of 19.73 and 23.77°. The addition of PEG 4000 resulted in the sharp and strong peaks, and this corresponds to the increasing crystallinity of the PLA matrix. Further, these changes were also observed at 2θ of 27.41° as weak curves. The intensities of the peaks increased by increasing the amount of PEG 4000.^24^

## 4 Heat Storage/Release Properties of PCM Nanofibers

**Scheme 1:**
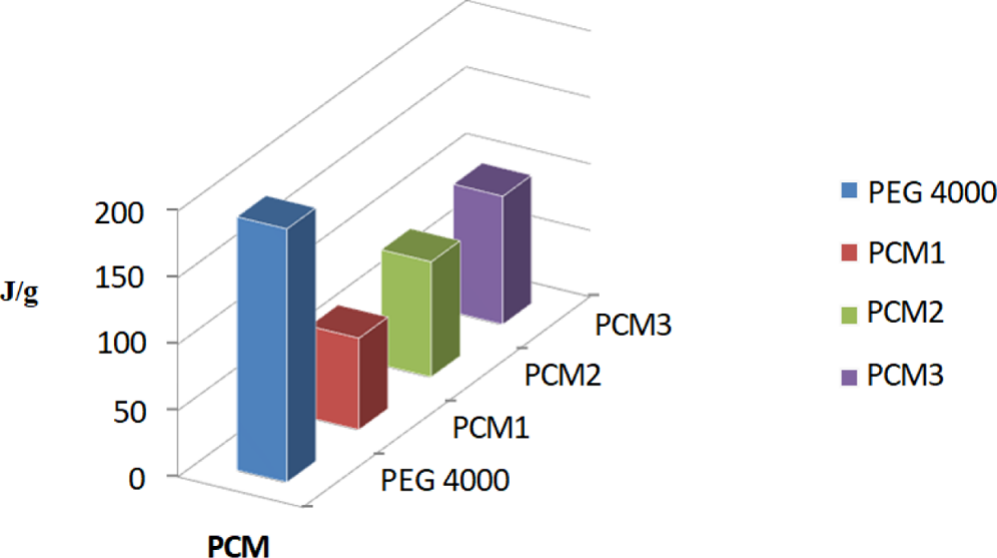
Latent Heat Storage Energy Value of PCM Nanofibers

According to DSC data of PCM composites, the lowest latent heat storage melting enthalpy is 69.06 J/g and the highest is 96.39 J/g (Figure 5b,d). PEG 4000 stored 190.8 J/g of latent heat energy at 60.86 °C (*T*_peak_) (Scheme 1 and Figure 5a),^23^ but PCM1, PCM2, and PCM3 stored latent heat energies 69.06, 86.76, and 96.39 J/g, respectively, and PCMs have significant thermal storage density.

## 5 Conclusions

Among the successfully produced DEO and PEG 4000 loaded PLA-based composite nanofibers, PCM3 has the highest latent heat storage energy. PEG-based PCMs degrade at high temperatures. PLA and DEO did not significantly change the thermal stability of PEG 4000. The incorporation of PEG 4000 into PLA affected the crystallinity of the PLA matrix. The antibacterial property of DEO may provide functional properties to the nanofibers. Antimicrobial smart packaging material PCMs can ensure that foods are transported safely and hot for a certain period. PCM nanofibers can release stored latent heat energy into the environment when the ambient temperature decreases or increases. With these features, PCM nanofibers can make significant contributions to medical, textile, and hot food logistics as an environmentally friendly, economical, and smart material.
